# Expression of serine/glycine metabolism-related proteins is different according to the thyroid cancer subtype

**DOI:** 10.1186/s12967-016-0915-8

**Published:** 2016-06-08

**Authors:** Woo Young Sun, Hye Min Kim, Woo-Hee Jung, Ja Seung Koo

**Affiliations:** Department of Surgery, Daejeon St. Mary’s Hospital, The Catholic University of Korea College of Medicine, Daejeon, South Korea; Department of Pathology, Severance Hospital, Yonsei University College of Medicine, 50 Yonsei-ro, Seodaemun-gu, Seoul, 120-752 South Korea

**Keywords:** Glycine, Serine, Metabolism, Thyroid cancer

## Abstract

**Background:**

The aim of this study was to investigate the expression and clinical implications of proteins related to serine/glycine metabolism in different subtypes of thyroid cancer.

**Methods:**

Tissue microarray (TMA) was constructed with tissues from 557 thyroid cancers, consisting of 244 papillary thyroid carcinomas (PTC), 112 follicular carcinomas (FC), 70 medullary carcinomas (MC), 23 poorly differentiated carcinomas (PDC), and 8 anaplastic carcinomas (AC). Immunohistochemical staining of the serine/glycine metabolism-related molecules phosphoglycerate dehydrogenase (PHGDH), phosphoserine aminotransferase, (PSAT), phosphoserine phosphatase (PSPH), serine hydromethyl transferase (SHMT), and glycine decarboxylase (GLDC) was performed with the TMA blocks and the results were analyzed together with clinicopathologic parameters.

**Results:**

The expression of serine/glycine metabolism-related proteins differed among thyroid cancer subtypes. The expression rate of PHGDH (p < 0.001), PSAT1 (p = 0.001), PSPH (p = 0.008), and tumoral SHMT1 (p < 0.001) was higher in PDC and PTC (78.3, 21.7, 21.7, 30.4 and 63.4, 18.6, 12.8, 31.4 %, respectively), and lowest in MC (15.7, 1.4, 0.0, 10.0 %). Stromal SHMT1 expression was highest in AC (62.5 %) and absent in all FC (p < 0.001). In PTC, positivity for PSPH (p = 0.041), tumoral SHMT1 (p = 0.018), and stromal SHMT1 (p < 0.001) expression was higher in the conventional type compared to follicular type (14.1 versus 2.5 %, 33.6 versus 15.0 %, 42.1 versus 10.0 %, respectively). BRAF V600E mutation was associated with a higher rate of PHGDH (p < 0.001), PSAT1 (p = 0.001), PSPH (p < 0.001), tumoral SHMT1 (p = 0.001), stromal SHMT1 (p < 0.001), and GLDC (p < 0.001) expression compared to non-mutant cases (73.5 versus 40.6 %, 23.1 versus 8.5 %, 17.6 versus 1.9 %, 37.0 versus 18.9 %, 45.8 versus 21.7 %, 21.8 versus 6.6 %, respectively). In univariate analysis, stromal SHMT1 expression was associated with shorter disease-free survival (p = 0.015) in follicular variant PTC, and GLDC positivity was associated with shorter overall survival (OS) in sclerotic stromal type (p = 0.002). In FC, minimally invasive type, PSPH positivity correlated with shorter OS (p = 0.045) and in MC, PHGDH positivity correlated with shorter OS (p = 0.034).

**Conclusion:**

The expression of serine/glycine metabolism-related proteins differs among different thyroid cancer types, with a higher rate of expression in PDC and PTC, and lower rate of expression in MC. In PTC, the rate of expression is lower in the follicular variant and higher in cases with BRAF V600E mutation.

**Electronic supplementary material:**

The online version of this article (doi:10.1186/s12967-016-0915-8) contains supplementary material, which is available to authorized users.

## Background

In malignant neoplasms a metabolic shift from oxidative phosphorylation in mitochondria to glycolysis occurs, a phenomenon known as the Warburg effect [1]. Neoplastic cells exhibiting increased glycolysis show increased levels of glycolytic intermediates. Recent studies reported that the metabolism of glycolytic intermediates is involved in tumorigenesis, for example through the glycine and serine metabolic pathways [2–5]. In the serine biosynthesis pathway, the 3-phosphoglycerate (3PG) that is generated in the glycolysis process is oxidized to 3-phosphohydroxypyruvate (pPYR) by phosphoglycerate dehydrogenase (PHGDH) [6]. pPYR is transaminated into phosphoserine (pSER) by phosphoserine aminotransferase (PSAT) [7], and pSER is dephosphorylated by phosphoserine phosphatase (PSPH) to produce serine [8]. In glycine metabolism, methylene-tetrahydrofolate is generated by glycine decarboxylase (GLDC), using glycine and tetrahydrofolate as substrates [9]. Serine metabolism and glycine metabolism are linked by serine hydromethyl transferase (SHMT), which catalyzes the reversible conversion of serine and glycine [10]. In previous studies, expression of PHGDH was reported to be increased in breast cancer and melanoma [3, 4], and expression of GLDC was reported to be increased in lung cancer [5], revealing a role of proteins related to serine/glycine metabolism in tumorigenesis.

Thyroid cancer is a common malignant neoplasm that occurs in 1 % of the population [11]. The most common subtype is papillary thyroid carcinoma (PTC), although there are several other subtypes, including follicular carcinoma (FC), medullary carcinoma (MC), poorly differentiated carcinoma (PDC), and anaplastic carcinoma (AC). These subtypes differ in cell origin, clinical manifestation, metastatic pattern, and clinical prognosis [12].

A representative mutation gene in thyroid cancer is the BRAF mutation gene. BRAF is a 75–100 kDa protein in the Raf kinase family that is the most potent activator of MEK kinase in the Ras-Raf-MEK-ERK pathway [2, 5]. The Ras-Raf-MEK-ERK pathway is abnormally activated in tumors and this aberrant ERK signaling is due to the BRAF mutation [4]. The BRAF mutation first reported in 2002 is a mutation in nucleotide 1796 in about 90 % that converts valine to glutamic acid in codon 599 (V599E; After NOMENCLATURE CHANGE, it was renamed to V600E) [13]. The BRAF V600E mutation appears in a variety of tumors found in cancers including thyroid papillary carcinoma (36–53 %), malignant melanoma (40–70 %), colorectal carcinoma (5–22 %), glioma (11 %), and ovary serous carcinoma (30 %) [3]. The BRAF mutation in PTC is associated with extra-thyroidal extension, advanced TNM stage, lymph node metastasis, multifocality, and recurrence [14].

It is thought that tumor metabolism, including serine and glycine metabolism, may differ among these subtypes, however there are few reports on this subject. The aim of this study was to investigate the expression and clinical implications of proteins related to serine/glycine metabolism according to thyroid cancer subtype.

## Methods

### Patient selection

Patients who were diagnosed with PTC and had surgical resection from January 2012 to December 2013 were enrolled in this study. Patients who were diagnosed with other subtypes of thyroid cancer from January 2000 to December 2014 were also enrolled. Patients who received preoperative chemotherapy were excluded. A thyroid pathologist (Koo JS) reviewed hematoxylin/eosin (H&E)-stained slides for all cases. Clinicopathologic data were obtained from the patients’ medical records and included age at diagnosis, disease recurrence, metastasis, current status, and length of follow-up. Tumor size, location (right or left lobe), extent (confined to the thyroid parenchyme or with extrathyroidal spread), and number of metastatic lymph nodes were also noted from review of the slides and the surgical pathology reports.

### Tissue microarray (TMA)

Representative areas were selected on H&E-stained slides and corresponding spots were marked on the surface of the matching paraffin blocks. Five-mm core biopsies were taken from selected areas and placed into a 5 × 4 recipient block. More than two tissue cores were extracted from each case to minimize extraction bias. Each tissue core was assigned a unique tissue microarray location number that was linked to a database containing other clinicopathologic data.

### Immunohistochemistry

Antibodies used for immunohistochemistry are listed in Additional file 1: Table S1. All immunohistochemistry was performed with formalin-fixed paraffin-embedded tissue sections using an automatic immunohistochemistry staining device (Benchmark XT, Ventana Medical System, Tucson, AZ, USA). Briefly, 5-µm thick formaldehyde-fixed paraffin-embedded tissue sections were transferred onto adhesive slides and dried at 62 °C for 30 min. Standard heat epitope retrieval was performed for 30 min in ethylene diaminetetraacetic acid, pH 8.0, in the autostainer. The samples were then incubated sequentially with primary antibodies, biotinylated anti-mouse immunoglobulin, peroxidase-labeled streptavidin (LSAB kit, DakoCytomation), and 3,30-diaminobenzidine. Negative control samples were processed without the primary antibody. Slides were counterstained with Harris hematoxylin. Positive control tissue was used as recommended by the manufacturer.

### Analysis of immunohistochemical staining

This study used the semi-quantitative ordinal scoring system based on visual inspection by pathologists. Immunohistochemical markers were assessed by light microscopy. The expression of serine/glycine metabolism-related proteins was evaluated semi-quantitatively as previously reported [15]. Tumor and stromal cell staining was scored as follows: 0, negative or weak immunostaining in <1 % of the tumor/stroma; 1, focal expression in 1–10 % of tumor/stroma; 2, positive in 11–50 % of tumor/stroma; 3, positive in 51–100 % of tumor/stroma. The evaluation was performed throughout the whole area of the tumor, and a score of 2 or higher was defined as positive. For the evaluation of BRAF V600E, tissue samples that had scores of 20 % or higher were regarded as positive [16]. Two pathologists (KJS and KHM) independently evaluated the expression according the scoring system and any mismatched result was further evaluated by a third pathologist (JW).

### Statistical analysis

Data were analyzed using SPSS for Windows, Version 12.0 (SPSS Inc., Chicago, IL, USA). For determination of statistical significance, Student’s *t* and Fisher’s exact tests were used for continuous and categorical variables, respectively. In the case of multiple comparisons, a corrected *p* value with the application of the Bonferroni multiple comparison procedure was used. Statistical significance was set to p < 0.05. Kaplan–Meier survival curves and log-rank statistics were employed to evaluate time to tumor recurrence and overall survival. Multivariate regression analysis was performed using the Cox proportional hazards model.

## Results

### Basal characteristics of thyroid cancer

Among 557 thyroid cancers, 344 (61.8 %) were PTC, 112 (20.1 %) were FC, 70 (12.6 %) were MC, 23 (4.1 %) were PDC, and 8 (1.4 %) were AC. Basal characteristics of PTC are summarized in Additional file 1: Table S2. PTC cases consisted of 304 (88.4 %) cases of conventional type and 40 (11.6 %) cases of follicular variant. Follicular variant PTC had a higher rate of expanding tumor margin (p = 0.002) than conventional type (32.5 versus 13.8 %). BRAF V600E mutation was present in 238 of PTCs (69.2 %) and was associated with an infiltrative tumor margin (87.8 %, p = 0.004) and conventional type (93.7 %, p < 0.001, Additional file 1: Table S2). The rate of follicular variant was only 6.3 % among cases with BRAF V600E. Follicular carcinoma consisted of 99 (88.4 %) cases of minimally invasive type and 13 (11.6 %) cases of widely invasive type, and the widely invasive type was associated with larger tumor size (>2.0 cm 100 versus 65.7 %, p = 0.040), vascular invasion (69.2 versus 37.4 %, p = 0.028), extrathyroidal involvement (53.8 versus 10.1 %, p < 0.001), and distant metastasis (38.5 versus 6.1 %, p = 0.003) compared to minimally invasive type (Additional file 1: Table S3). Basal characteristics of MC, PDC, and AC are summarized in Additional file 1: Table S4.

### Expression of serine/glycine metabolism-related proteins in thyroid cancer subtypes

**Fig. 1:**
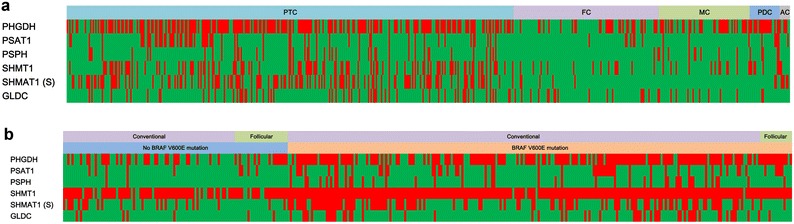
Heat map of expression of serine/glycine metabolism-related proteins in thyroid cancer (**a**) and papillary thyroid cancer (**b**). *Red*, positive; *green*, negative, *PTC* papillary thyroid carcinoma; *FC* follicular carcinoma; *MC* medullary carcinoma; *PDC* poorly differentiated carcinoma; *AC* anaplastic carcinoma; *PHGDH* phosphoglycerate dehydrogenase; *PSAT* phosphoserine aminotransferase; *PSPH* phosphoserine phosphatase; *SHMT* serine hydromethyl transferase; *GLDC* glycine decarboxylase; *S* stroma

**Fig. 2:**
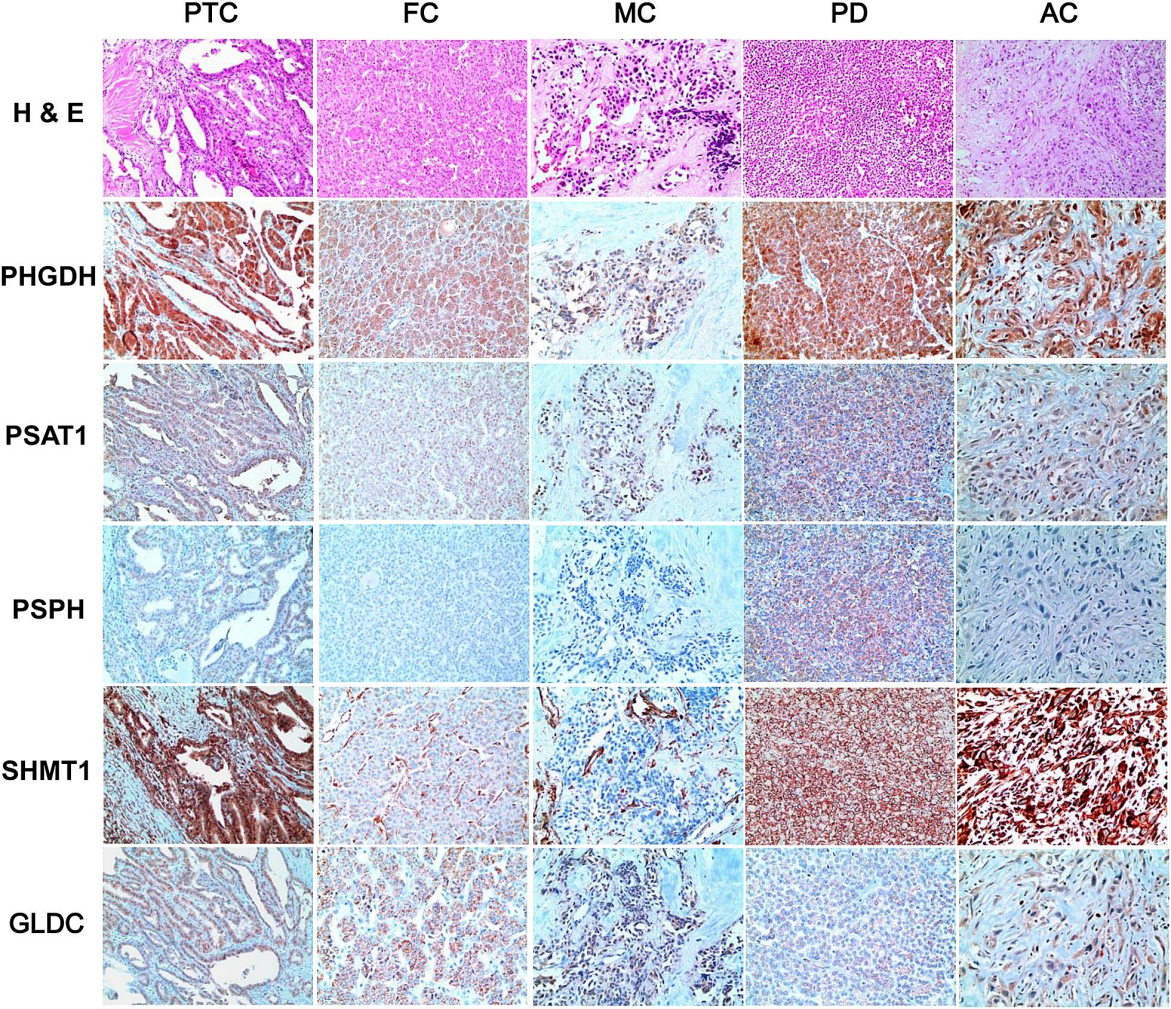
Expression of serine/glycine metabolism-related proteins in thyroid cancer. The expression of PHGDH, PSAT1, PSPH, tumoral SHMT1, and stromal SHMT1 is different according to cancer subtype. The expression of PHGDH, PSAT1, PSPH, and tumoral SHMT1 is higher in PDC and PTC, and lowest in MC. The expression of stromal SHMT1 is highest in AC and lowest in FC

The expression of serine/glycine metabolism-related proteins was investigated in all thyroid cancers. For the serine/glycine metabolism-related proteins, PHGDH had the highest expression rate (54.8 %) compared to lower expression rates of stromal SHMT1 (25.9 %), tumoral SHMT1 (25.5 %), GLDC (15.6 %), PSAT1 (14.4 %), and PSPH (10.8 %). Therefore, serine/glycine metabolism-related protein expression rate was low in most cases except for PHGDH (Table 1). The expression of PHGDH (p < 0.001), PSAT1 (p = 0.001), PSPH (p = 0.008), tumoral SHMT1 (p < 0.001), and stromal SHMT1 (p < 0.001) was different according to cancer subtype (Fig. 1a). The expression of PHGDH, PSAT1, PSPH, and tumoral SHMT1 was higher in PDC and PTC (78.3, 21.7, 21.7, 30.4 and 63.4, 18.6, 12.8, 31.4 %, respectively), and lowest in MC (15.7, 1.4, 0.0, 10.0 %). The expression of stromal SHMT1 was highest in AC (62.5 %) and lowest in FC (0.0 %) (Table 1; Fig. 2).

**Table 1:** Expression of serine/glycine metabolism-related proteins according to the subtype of thyroid cancer

Parameters	Total n = 557 (%)	PTC n = 344 (%)	FC n = 112 (%)	MC n = 70 (%)	PDC n = 23 (%)	AC n = 8 (%)	P value
PHGDH in tumor							<*0.001*
Negative	252 (45.2)	126 (36.6)	58 (51.8)	59 (84.3)	5 (21.7)	4 (50.0)	
Positive	305 (54.8)	218 (63.4)	54 (48.2)	11 (15.7)	18 (78.3)	4 (50.0)	
PSAT1 in tumor							*0.001*
Negative	477 (85.6)	280 (81.4)	103 (92.0)	69 (98.6)	18 (78.3)	7 (87.5)	
Positive	80 (14.4)	64 (18.6)	9 (8.0)	1 (1.4)	5 (21.7)	1 (12.5)	
PSPH in tumor							*0.008*
Negative	497 (89.2)	300 (87.2)	101 (90.2)	70 (100.0)	18 (78.3)	8 (100.0)	
Positive	60 (10.8)	44 (12.8)	11 (9.8)	0 (0.0)	5 (21.7)	0 (0.0)	
SHMT1 in tumor							<*0.001*
Negative	415 (74.5)	236 (68.6)	94 (83.9)	63 (90.0)	16 (69.6)	6 (75.0)	
Positive	142 (25.5)	108 (31.4)	18 (16.1)	7 (10.0)	7 (30.4)	2 (25.0)	
SHMT1 in stroma							<*0.001*
Negative	413 (74.1)	212 (61.6)	112 (100.0)	65 (92.9)	21 (91.3)	3 (37.5)	
Positive	144 (25.9)	132 (38.4)	0 (0.0)	5 (7.1)	2 (8.7)	5 (62.5)	
GLDC in tumor							0.529
Negative	470 (84.4)	285 (82.8)	96 (85.7)	60 (85.7)	21 (91.3)	8 (100.0)	
Positive	87 (15.6)	59 (17.2)	16 (14.3)	10 (14.3)	2 (8.7)	0 (0.0)	

*PTC* papillary thyroid carcinoma; *FC* follicular carcinoma; *MC* medullary carcinoma; *PDC* poorly differentiated carcinoma; *AC* anaplastic carcinoma; *PHGDH* phosphoglycerate dehydrogenase; *PSAT* phosphoserine aminotransferase; *PSPH* phosphoserine phosphatase; *SHMT* serine hydromethyl transferase; *GLDC* glycine decarboxylase

Italic values represent p < 0.05

**Fig. 3:**
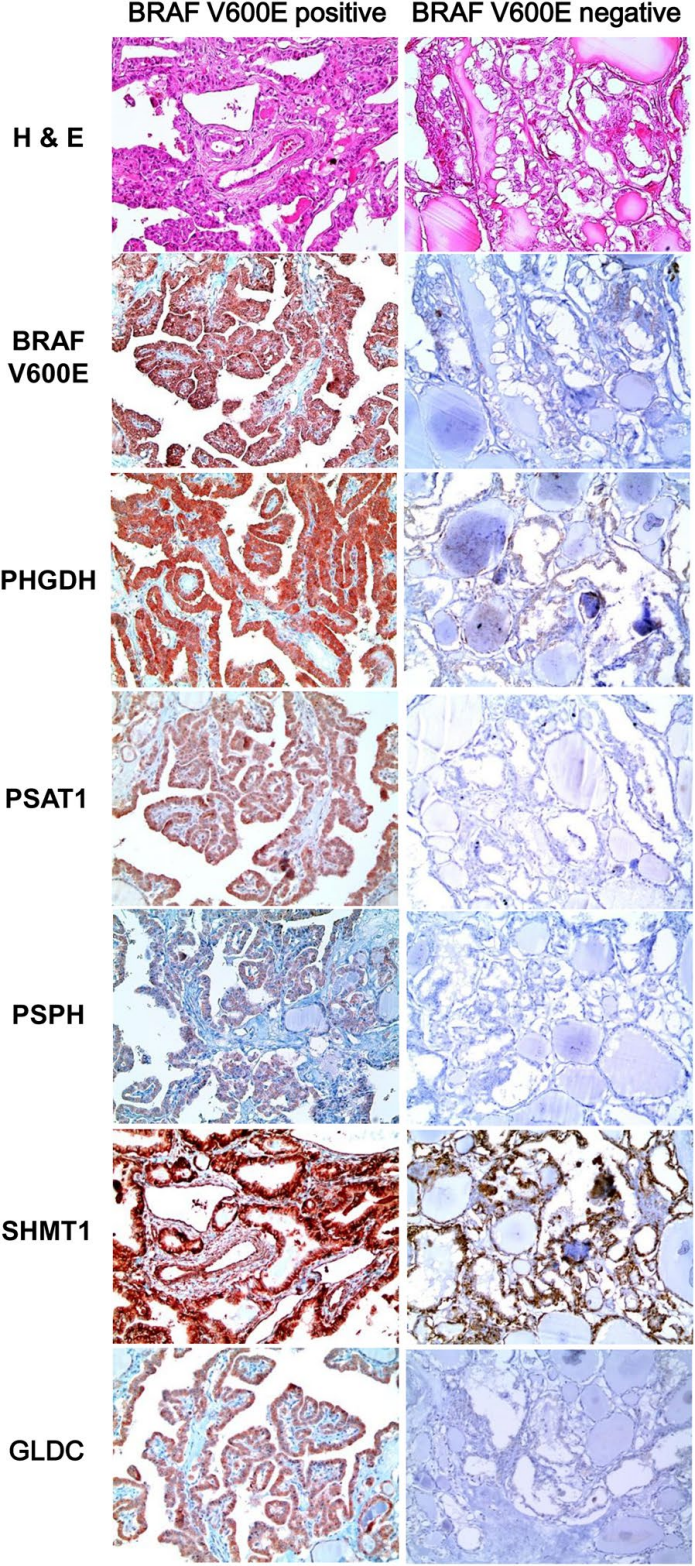
Expression of serine/glycine metabolism-related proteins according to the status of BRAF V600E mutation in papillary carcinoma. BRAF V600E mutation status was associated with the expression of PHGDH, PSAT1, PSPH, tumoral SHMT1, stromal SHMT1, and GLDC, with higher expression of serine/glycine metabolism-related proteins in BRAF V600E mutation-positive cases

Among PTCs, the expression of PSPH (p = 0.041), tumoral SHMT1 (p = 0.018), and stromal SHMT1 (p < 0.001) differed according to histologic subtypes, with a higher rate of expression in the conventional type compared to follicular type (14.1 versus 2.5 %, 33.6 versus 15.0 %, 42.1 versus 10.0 %, respectively) (Table 2; Fig. 1b). BRAF V600E mutation status was associated with the expression of PHGDH (p < 0.001), PSAT1 (p = 0.001), PSPH (p < 0.001), tumoral SHMT1 (p = 0.001), stromal SHMT1 (p < 0.001), and GLDC (p < 0.001), with higher expression of serine/glycine metabolism-related proteins in BRAF V600E mutation-positive cases compared to non-mutant cases (73.5 versus 40.6 %, 23.1 versus 8.5 %, 17.6 versus 1.9 %, 37.0 versus 18.9 %, 45.8 versus 21.7 %, 21.8 versus 6.6 %, respectively) (Table 2; Fig. 3).

**Table 2:** Expression of glutamine metabolism-related proteins according to the histologic subtype of PTC

Parameters	Total n = 344 (%)	Histologic subtype		p value	BRAF V600E mutation status		p value
		Conventional type n = 304 (%)	Follicular variant n = 40 (%)		No mutation n = 106 (%)	Mutation n = 238 (%)	
PHGDH in tumor				0.564			<*0.001*
Negative	126 (36.6)	113 (37.2)	13 (32.5)		63 (59.4)	63 (26.5)	
Positive	218 (63.4)	191 (62.8)	27 (67.5)		43 (40.6)	175 (73.5)	
PSAT1 in tumor				0.501			*0.001*
Negative	280 (81.4)	249 (81.9)	31 (77.5)		97 (91.5)	183 (76.9)	
Positive	64 (18.6)	55 (18.1)	9 (22.5)		9 (8.5)	55 (23.1)	
PSPH in tumor				*0.041*			<*0.001*
Negative	300 (87.2)	261 (85.9)	39 (97.5)		104 (98.1)	196 (82.4)	
Positive	44 (12.8)	43 (14.1)	1 (2.5)		2 (1.9)	42 (17.6)	
SHMT1 in tumor				*0.018*			*0.001*
Negative	236 (68.6)	202 (66.4)	34 (85.0)		86 (81.1)	150 (63.0)	
Positive	108 (31.4)	102 (33.6)	6 (15.0)		20 (18.9)	88 (37.0)	
SHMT1 in stroma				<*0.001*			<*0.001*
Negative	212 (61.6)	176 (57.9)	36 (90.0)		83 (78.3)	129 (54.2)	
Positive	132 (38.4)	128 (42.1)	4 (10.0)		23 (21.7)	109 (45.8)	
GLDC in tumor				0.116			<*0.001*
Negative	285 (82.8)	248 (81.6)	37 (92.5)		99 (93.4)	186 (78.2)	
Positive	59 (17.2)	56 (18.4)	3 (7.5)		7 (6.6)	52 (21.8)	

*PTC* papillary thyroid carcinoma; *PHGDH* phosphoglycerate dehydrogenase; *PSAT* phosphoserine aminotransferase; *PSPH* phosphoserine phosphatase; *SHMT* serine hydromethyl transferase; *GLDC* glycine decarboxylase

Italic values represent p < 0.05

Among follicular neoplasms, the expression of PSAT1 was higher in follicular adenoma compared to FC (24.2 versus 8.0 %, p = 0.001, Table 3). There was no difference in serine/glycine metabolism-related protein expression between the minimally invasive and widely invasive type of FC (Table 4).

**Table 3:** Expression of serine/glycine metabolism-related proteins in follicular neoplasm

Parameters	Total n = 264 (%)	Follicular adenoma n = 152 (%)	Follicular carcinoma n = 112 (%)	p value
PHGDH in tumor				0.274
Negative	147 (55.7)	89 (58.6)	58 (51.8)	
Positive	117 (44.3)	63 (41.4)	54 (48.2)	
PSAT1 in tumor				*0.001*
Negative	219 (82.6)	116 (75.8)	103 (92.0)	
Positive	46 (17.4)	37 (24.2)	9 (8.0)	
PSPH in tumor				0.328
Negative	244 (92.1)	143 (93.5)	101 (90.2)	
Positive	21 (7.9)	10 (6.5)	11 (9.8)	
SHMT1 in tumor				0.137
Negative	211 (79.6)	117 (76.5)	94 (83.9)	
Positive	54 (20.4)	36 (23.5)	18 (16.1)	
GLDC in tumor				0.262
Negative	234 (88.3)	138 (90.2)	96 (85.7)	
Positive	31 (11.7)	15 (9.8)	16 (14.3)	

*PHGDH* phosphoglycerate dehydrogenase; *PSAT* phosphoserine aminotransferase; *PSPH* phosphoserine phosphatase; *SHMT* serine hydromethyl transferase; *GLDC* glycine decarboxylase

Italic value represents p < 0.05

**Table 4:** Expression of serine/glycine metabolism-related proteins according to the histologic subtype of follicular carcinoma

Parameters	Total n = 112 (%)	FC, minimally invasive type n = 99 (%)	FC, widely invasive type n = 13 (%)	p value
PHGDH in tumor				0.454
Negative	58 (51.8)	50 (50.5)	8 (61.5)	
Positive	54 (48.2)	49 (49.5)	5 (38.5)	
PSAT1 in tumor				1.000
Negative	103 (92.0)	91 (91.9)	12 (92.3)	
Positive	9 (8.0)	8 (8.1)	1 (7.7)	
PSPH in tumor				0.357
Negative	101 (90.2)	88 (88.9)	13 (100.0)	
Positive	11 (9.8)	11 (11.1)	0 (0.0)	
SHMT1 in tumor				0.689
Negative	94 (83.9)	82 (82.8)	12 (92.3)	
Positive	18 (16.1)	17 (17.2)	1 (7.7)	
GLDC in tumor				1.000
Negative	96 (85.7)	85 (85.9)	11 (84.6)	
Positive	16 (14.3)	14 (14.1)	2 (15.4)	

*PHGDH* phosphoglycerate dehydrogenase; *PSAT* phosphoserine aminotransferase; *PSPH* phosphoserine phosphatase; *SHMT* serine hydromethyl transferase; *GLDC* glycine decarboxylase

### Correlation between the expression of serine/glycine metabolism-related proteins and clinicopathologic factors

**Fig. 4:**
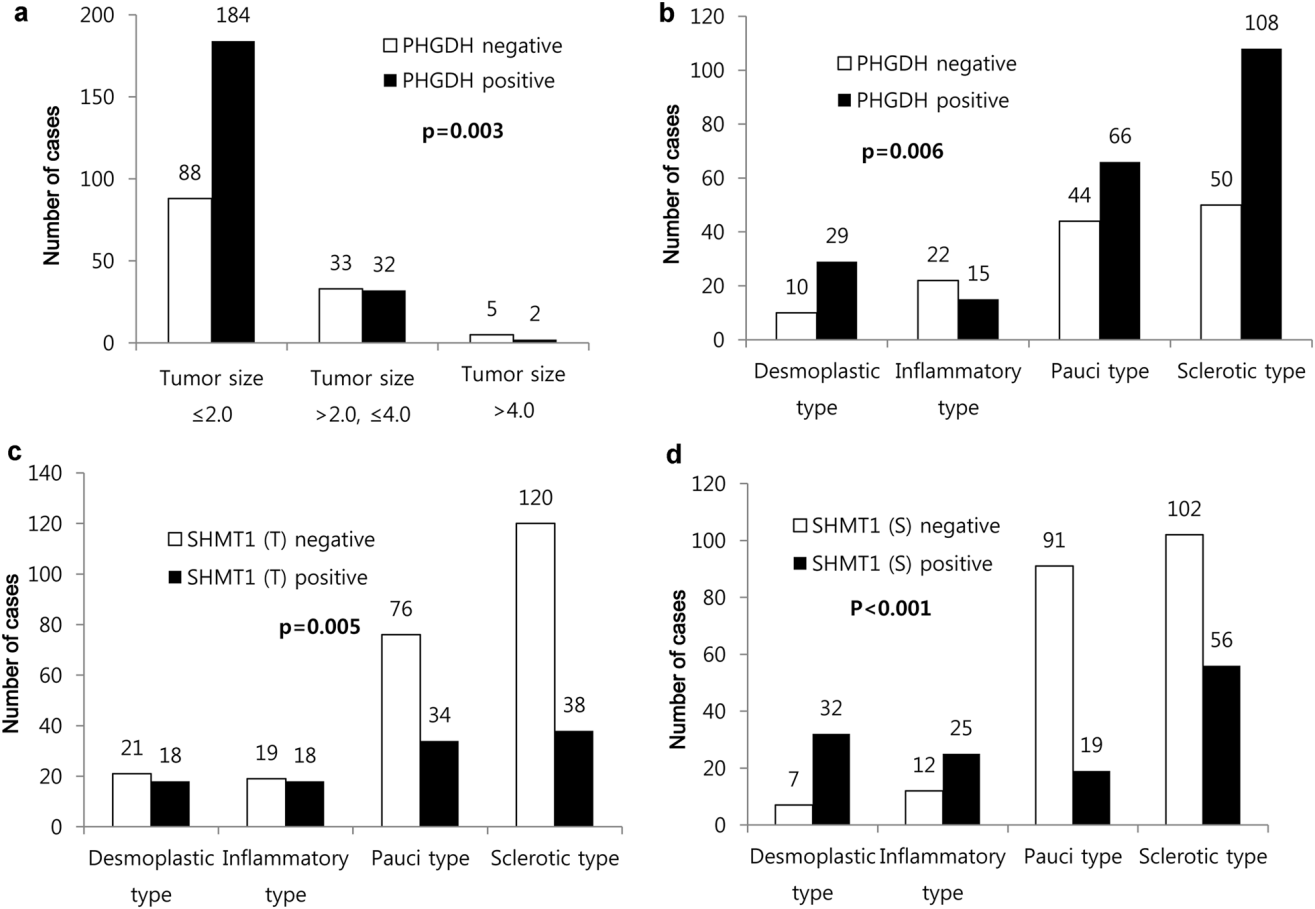
Correlation between the expression of serine/glycine metabolism-related proteins and clinicopathologic factors in papillary carcinoma. **a** In PTC, PHGDH expression is associated with tumor size, with an increased tumor size in PHGDH-positive cases. **b** Inflammatory type shows a lower rate of PHGDH expression compared to other subtypes, and desmoplastic type and inflammatory type have a higher rate of tumoral (**c**) and stromal **d** SHMT1 expression compared to pauci type and sclerotic type

Correlations between the expression of serine/glycine metabolism-related proteins and clinicopathologic factors of thyroid cancers were analyzed. In PTC, PHGDH expression was associated with tumor size (p = 0.003), with an increased tumor size in PHGDH-positive cases. In addition, stromal histologic type of PTC was associated with PHGDH (p = 0.006), tumoral SHMT1 (p = 0.005), and stromal SHMT1 (p < 0.001) expression. Inflammatory type showed a lower rate of PHGDH expression compared to other subtypes, and desmoplastic type and inflammatory type had a higher rate of tumoral and stromal SHMT1 expression compared to pauci type and sclerotic type (Fig. 4).

### Impact of the expression of serine/glycine metabolism-related proteins on patient prognosis

**Fig. 5:**
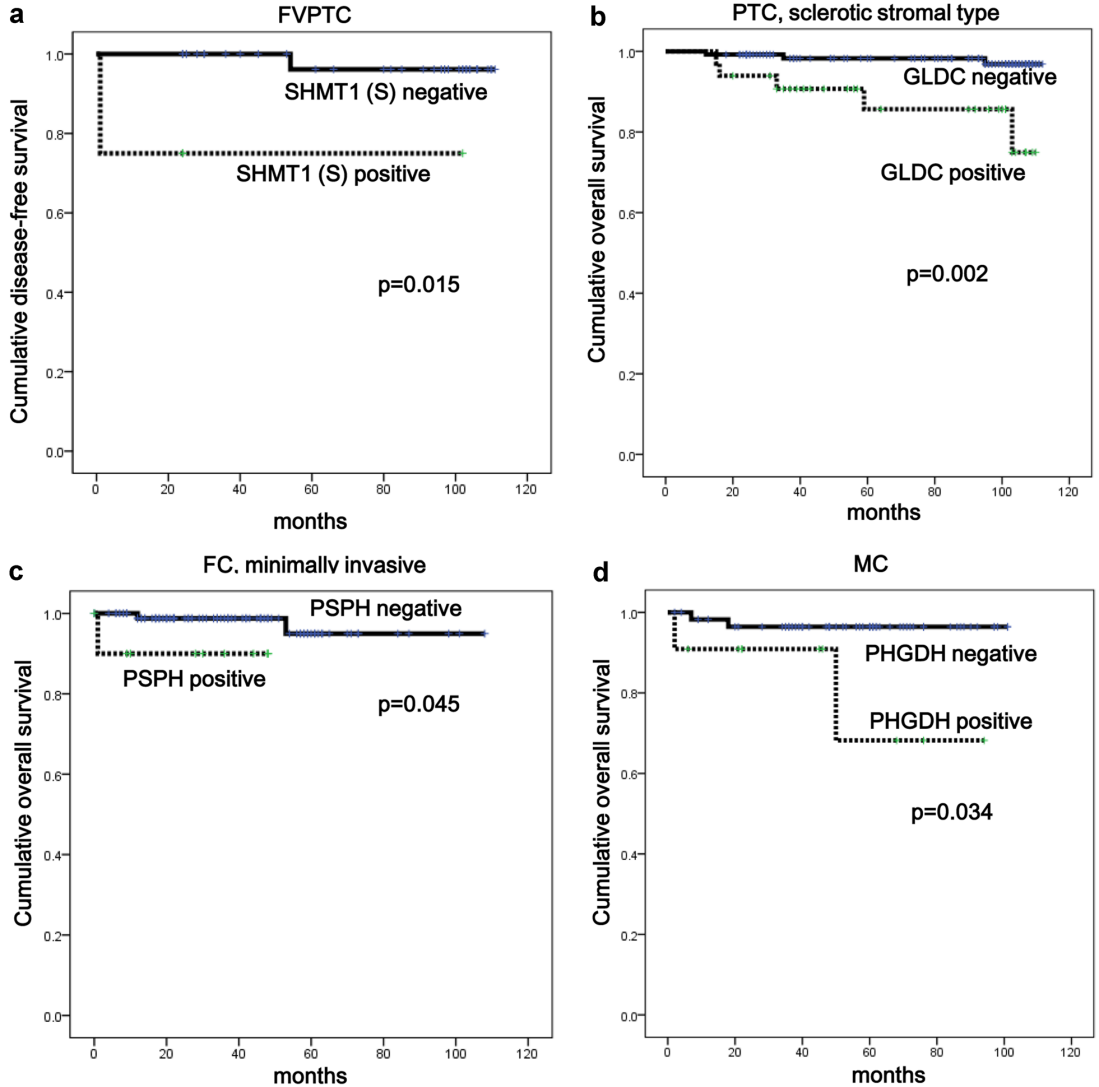
Impact of the expression of serine/glycine metabolism-related proteins on patient prognosis. **a** For follicular variant PTC, the expression of stromal SHMT1 is associated with shorter disease-free survival. **b** In the sclerotic stromal type, GLDC positivity is associated with shorter overall survival. **c** PSPH positivity is associated with shorter OS in minimally invasive type FC. **d** PHGDH positivity correlated with shorter OS in MC

In analysis of the impact of the expression of serine/glycine metabolism-related proteins on patient prognosis, the expression of serine/glycine metabolism-related proteins did not have an association with prognosis for overall PTC (Table 5). However, for follicular variant PTC the expression of stromal SHMT1 was associated with shorter disease-free survival (DFS; p = 0.015), and in the sclerotic stromal type, GLDC positivity was associated with shorter overall survival (OS; p = 0.002). PSPH positivity was associated with shorter OS in minimally invasive type FC (p = 0.045) and PHGDH positivity correlated with shorter OS in MC (p = 0.034, and Fig. 5).

**Table 5:** Univariate analysis of the impact of serine/glycine-related protein expression in papillary thyroid carcinoma on disease-free and overall survival by the log-rank test

Parameter	Number of patients^a^/recurrence/death	Disease-free survival		Overall survival	
		Mean survival (95 % CI) months	p value	Mean survival (95 % CI) months	p value
PHGDH in tumor			0.790		0.806
Negative	126/6/6	105 (101–108)		106 (103–109)	
Positive	218/12/12	106 (104–109)		107 (105–110)	
PSAT1 in tumor			0.159		0.231
Negative	280/17/13	106 (103–109)		108 (106–110)	
Positive	64/1/5	108 (105–111)		103 (97–108)	
PSPH in tumor			0.606		0.221
Negative	300/15/14	107 (105–109)		108 (106–110)	
Positive	44/3/4	103 (96–110)		103 (96–109)	
SHMT1 in tumor			0.498		0.248
Negative	236/11/10	107 (105–110)		108 (106–110)	
Positive	108/7/8	104 (100–109)		105 (101–109)	
SHMT1 in stroma			0.655		0.684
Negative	212/12/10	106 (103–109)		108 (106–110)	
Positive	132/6/8	107 (104–111)		107 (103–110)	
GLDC in tumor			0.516		0.059
Negative	285/14/12	107 (105–109)		108 (106–110)	
Positive	59/4/6	104 (97–110)		103 (97–109)	

*CI* confidence interval; *PHGDH* phosphoglycerate dehydrogenase; *PSAT* phosphoserine aminotransferase; *PSPH* phosphoserine phosphatase; *SHMT* serine hydromethyl transferase; *GLDC* glycine decarboxylase

^a^Cases of anaplastic carcinoma were excluded

## Discussion

In this study, we investigated the expression of proteins related to serine/glycine metabolism in thyroid cancers and found differences in expression according to the different types of thyroid cancer. In general, the rate of serine/glycine metabolism-related protein expression was higher in PDC and PTC, and lower in MC. As there are no previous studies regarding serine/glycine metabolism in thyroid cancer a direct comparison cannot be made, however MC is a tumor that originates from C-cells whereas other thyroid neoplasms originate from follicular cells, so they might be expected to have different metabolic features. The results of our study suggest that C-cell origin MC may have lower serine/glycine metabolic activity, although further study is required to examine this finding. The present study showed that stromal SHMT1 expression was highest in AC. In previous studies of breast cancer, it was reported that serine/glycine metabolism-related proteins were expressed not only in tumor cells but also in stromal cells [17, 18]. Moreover, glycolysis [19–21] and glutamine metabolism were reported to mediate metabolic interactions between cancer cells and stromal cells such as cancer-associated fibroblasts (CAFs) [20, 22–25]. Metabolic interactions between cancer cells and CAFs promote tumor cell proliferation and growth, suggesting that serine/glycine metabolic activity in both tumor and stroma contributes to tumor aggressiveness in AC.

We observed a higher rate of serine/glycine metabolism-related protein expression in cases of PTC with the BRAF V600E mutation. In meta-analysis studies, the BRAF V600E mutation is reported to be associated with extra-thyroidal extension, advanced TNM stage, lymph node metastasis, multifocality, and recurrence [14], suggesting that PTC with a BRAF V600E mutation has more aggressive tumor biology. In general, higher metabolic activity is related to increased tumor aggressiveness [26–28], and higher expression levels of proteins related to serine/glycine metabolism might be expected in BRAF V600E-positive cases. In addition, PTC with the BRAF V600E mutation is reported to have enhanced glucose metabolism [29]. Since serine metabolism initiates from 3PG, which is generated in the glycolysis pathway, increased serine metabolism might be expected when glycolysis is increased.

A clinical implication of this study is the potential application of targeted therapy against the serine/glycine metabolism pathway. Preclinical studies are ongoing to identify drugs that target multiple sites of one-carbon metabolism, such as GLDC, PSAT, PSPH, and PHGDH, as a novel therapeutic approach [30, 31].

## Conclusion

The expression of serine/glycine metabolism-related proteins differs among different thyroid cancer types, with a higher rate of expression in PDC and PTC, and a lower rate of expression in MC. In PTC, the rate of serine/glycine metabolism-related protein expression is lower in the follicular variant, and higher in cases with the BRAF V600E mutation.

## Additional file

10.1186/s12967-016-0915-8 Source, clone, and dilution of antibodies used in this study. **Table S2.** Basal characteristics of thyroid papillary carcinoma. **Table S3.** Basal characteristics of thyroid follicular carcinoma. **Table S4.** Basal characteristics of thyroid medullary carcinoma, poorly differentiated carcinoma, and anaplastic carcinoma.

## Abbreviations

3PG: 3-phosphoglycerate
pPYR: phosphohydroxypyruvate
PHGDH: phosphoglycerate dehydrogenase
pSER: phosphoserine
PSAT: phosphoserine aminotransferase
PSPH: phosphoserine phosphatase
GLDC: glycine decarboxylase
SHMT: serine hydromethyl transferase
PTC: papillary thyroid carcinoma
FC: follicular carcinoma
MC: medullary carcinoma
PDC: poorly differentiated carcinoma
AC: anaplastic carcinoma
H&E: hematoxylin and eosin
CAF: cancer-associated fibroblast
