# Supplementary material for: Expression of serine/glycine metabolism-related proteins is different according to the thyroid cancer subtype
Source: J Transl Med. 2016 Jun 8;14:168. doi: 10.1186/s12967-016-0915-8 (PMC4898323; doi:10.1186/s12967-016-0915-8)
Supplement: Supplementary file 1 — 10.1186/s12967-016-0915-8 Source, clone, and dilution of antibodies used in this study. Table S2. Basal characteristics of thyroid papillary carcinoma. Table S3. Basal characteristics of thyroid follicular carcinoma. Table S4. Basal characteristics of thyroid medullary carcinoma, poorly differentiated carcinoma, and anaplastic carcinoma. [file 12967_2016_915_MOESM1_ESM.doc]

| Supplementary table 1. Source, clone, and dilution of antibodies used in this study | | | |
| --- | --- | --- | --- |
| **antibody** | **company** | **clone** | **dilution** |
| *serine/glycine metabolism related* |  |  |  |
| PHGDH | Abcam, Cambridge, UK | Polyclonal | 1:100 |
| PSAT1 | Abcam, Cambridge, UK | Polyclonal | 1:100 |
| PSPH | Abcam, Cambridge, UK | Polyclonal | 1:100 |
| SHMT1 | Abcam, Cambridge, UK | Polyclonal | 1:100 |
| GLDC | Abcam, Cambridge, UK | Polyclonal | 1:100 |

| Supplementary table 2. Basal characteristics of thyroid papillary carcinoma | | | | | | | |
| --- | --- | --- | --- | --- | --- | --- | --- |
| Parameters | Total  N=344 (%) | Histologic subtype | | p-value | BRAF V600E mutation status | | p-value |
|  |  | Conventional type  n= 304 (%) | Follicular variant  n= 40 (%) |  | No mutation  n= 106 (%) | Mutation  n= 238 (%) |  |
| Age (years) |  |  |  | 0.741 |  |  | 0.089 |
| <45 | 155 (45.1) | 136 (44.7) | 19 (47.5) |  | 55 (51.9) | 100 (42.0) |  |
| ≥45 | 189 (54.9) | 168 (55.3) | 21 (52.5) |  | 51 (48.1) | 138 (58.0) |  |
| Sex |  |  |  | 0.969 |  |  | 0.235 |
| Male | 68 (19.8) | 60 (19.7) | 8 (20.0) |  | 25 (23.6) | 43 (18.1) |  |
| Female | 276 (80.2) | 244 (80.3) | 32 (80.0) |  | 81 (76.4) | 195 (81.9) |  |
| Tumor size (cm) |  |  |  | 0.951 |  |  | 0.506 |
| ≤2.0 | 272 (79.1) | 240 (78.9) | 32 (80.0) |  | 80 (75.5) | 192 (80.7) |  |
| >2.0, ≤4.0 | 65 (18.9) | 58 (19.1) | 7 (17.5) |  | 23 (21.7) | 42 (17.6) |  |
| >4.0 | 7 (2.0) | 6 (2.0) | 1 (2.5) |  | 3 (2.8) | 4 (1.7) |  |
| Tumor margin |  |  |  | **0.002** |  |  | **0.004** |
| Infiltrative | 289 (84.0) | 262 (67.5) | 27 (67.5) |  | 80 (75.5) | 209 (87.8) |  |
| Expanding | 55 (16.0) | 42 (13.8) | 13 (32.5) |  | 26 (24.5) | 29 (12.2) |  |
| Tumor extension |  |  |  | 0.330 |  |  | 0.177 |
| Intrathyroidal | 106 (30.8) | 91 (29.9) | 15 (37.5) |  | 38 (35.8) | 68 (28.6) |  |
| Extrathyroidal | 238 (69.2) | 213 (70.1) | 25 (62.5) |  | 68 (64.2) | 170 (71.4) |  |
| Histologic subtype |  |  |  |  |  |  | **<0.001** |
| Conventional |  |  |  |  | 81 (76.4) | 223 (93.7) |  |
| Follicular |  |  |  |  | 25 (23.6) | 15 (6.3) |  |
| LN metastasis |  |  |  | 0.175 |  |  | 0.075 |
| No | 138 (40.1) | 118 (38.8) | 20 (50.0) |  | 50 (47.2) | 88 (37.0) |  |
| Yes | 206 (59.9) | 186 (61.2) | 20 (50.0) |  | 56 (52.8) | 150 (63.0) |  |
| Distant metastasis |  |  |  | 0.944 |  |  | 0.446 |
| No | 326 (94.8) | 288 (94.7) | 38 (95.0) |  | 99 (93.4) | 227 (95.4) |  |
| Yes | 18 (5.2) | 16 (5.3) | 2 (5.0) |  | 7 (6.6) | 11 (4.6) |  |

| Supplementary table 3. Basal characteristics of thyroid follicular carcinoma | | | | |
| --- | --- | --- | --- | --- |
| Parameters | Total  N=112 (%) | FC, minimally invasive type  n=99 (%) | FC, widely invasive type  n=13 (%) | p-value |
| Age (years) |  |  |  | 0.255 |
| <45 | 51 (45.5) | 47 (47.5) | 4 (30.8) |  |
| ≥45 | 61 (54.5) | 52 (52.5) | 9 (69.2) |  |
| Sex |  |  |  | 0.233 |
| Male | 28 (25.0) | 23 (23.2) | 5 (38.5) |  |
| Female | 84 (75.0) | 76 (76.8) | 8 (61.5) |  |
| Tumor size (cm) |  |  |  | 0.040 |
| ≤2.0 | 34 (30.4) | 34 (34.3) | 0 (0.0) |  |
| >2.0, ≤4.0 | 49 (43.8) | 41 (41.4) | 8 (61.5) |  |
| >4.0 | 29 (25.9) | 24 (24.2) | 5 (38.5) |  |
| Capsular invasion |  |  |  | 0.147 |
| No | 14 (12.5) | 14 (14.1) | 0 (0.0) |  |
| Yes | 98 (87.5) | 85 (85.9) | 13 (100.0) |  |
| Vascular invasion |  |  |  | 0.028 |
| No | 66 (58.9) | 62 (62.6) | 4 (30.8) |  |
| Yes | 46 (41.1) | 37 (37.4) | 9 (69.2) |  |
| Tumor extension |  |  |  | <0.001 |
| Intrathyroidal | 95 (84.8) | 89 (89.9) | 6 (46.2) |  |
| Extrathyroidal | 17 (15.2) | 10 (10.1) | 7 (53.8) |  |
| LN metastasis |  |  |  | 0.220 |
| No | 110 (98.2) | 98 (99.0) | 12 (92.3) |  |
| Yes | 2 (1.8) | 1 (1.0) | 1 (7.7) |  |
| Distant metastasis |  |  |  | 0.003 |
| No | 101 (90.2) | 93 (93.9) | 8 (61.5) |  |
| Yes | 11 (9.8) | 6 (6.1) | 5 (38.5) |  |

| Supplementary table 4. Basal characteristics of thyroid medullary carcinoma | | | |
| --- | --- | --- | --- |
| Parameters | MC, n= 70 (%) | PDC, n= 23 (%) | AC, n= 8 (%) |
| Age (years) |  |  |  |
| <45 | 21 (30.0) | 4 (17.4) | 0 (0.0) |
| ≥45 | 49 (70.0) | 19 (82.6) | 8 (100.0) |
| Sex |  |  |  |
| Male | 22 (31.4) | 10 (43.5) | 1 (12.5) |
| Female | 48 (68.6) | 13 (56.5) | 7 (87.5) |
| Tumor size (cm) |  |  |  |
| ≤2.0 | 53 (75.7) | 8 (34.8) | 0 (0.0) |
| >2.0, ≤4.0 | 14 (20.0) | 9 (39.1) | 1 (12.5) |
| >4.0 | 3 (4.3) | 6 (26.1) | 7 (87.5) |
| Tumor margin |  |  |  |
| Infiltrative | 45 (64.3) | 17 (73.9) | 8 (100.0) |
| Expanding | 25 (35.7) | 6 (26.1) | 0 (0.0) |
| Tumor extension |  |  |  |
| Intrathyroidal | 52 (74.3) | 11 (47.8) | 0 (0.0) |
| Extrathyroidal | 18 (25.7) | 12 (52.2) | 8 (100.0) |
| LN metastasis |  |  |  |
| No | 47 (67.1) | 22 (95.7) | 4 (50.0) |
| Yes | 23 (32.9) | 1 (4.3) | 4 (50.0) |
| Distant metastasis |  |  |  |
| No | 67 (95.7) | 16 (69.6) | 8 (100.0) |
| Yes | 3 (4.3) | 7 (30.4) | 0 (0.0) |
